# Gender, Addiction, and Removal of Children Into Care

**DOI:** 10.3389/fpsyt.2022.887660

**Published:** 2022-06-02

**Authors:** Lynda Russell, Ruchika Gajwani, Fiona Turner, Helen Minnis

**Affiliations:** Mental Health and Wellbeing, Institute of Health and Wellbeing, University of Glasgow, Glasgow, United Kingdom

**Keywords:** addiction, mothers, child removal, suicide, gender

## Abstract

**Introduction:**

Parental addiction can result in harm to children and removal of children by the Local Authority. Less is known about the impact of removal of children on their parents and whether gender has a role in this process.

**Methods:**

Data on 736 service users were obtained from the caseloads of 8 nurses and 12 social care workers from an Alcohol and Drug Recovery Service in Scotland. Gender differences in prevalence/patterns of child removal, associations between child removal and parental factors and the relationship between removal and suicidality were examined.

**Results:**

Mothers were more likely to have had one or more children removed compared to fathers (56.6 vs. 17.7%; *p* < 0.001) and were more likely to have a series of individual child removals (22.5 vs. 4.3%; *p* = 0.014). In addition to female gender, younger age, drug use, mental health and suicide attempts were also associated with child removal. Mothers who had children removed and women who were not mothers were more likely to have made an attempt to end their lives than women who had children but had not had them removed.

**Conclusion:**

Gender differences were apparent in prevalence and patterns of child removal. Mothers were six times more likely to have children removed compared to fathers. Child removal occurred alongside other risk factors suggesting that families need holistic support for their multiple areas of need. Services should be aware of the link between child removal and suicide and provide additional support to mothers during and after removal.

## Introduction

Parental addiction^1^ has been associated with harm to children (1–3). In a Scottish context, drug or alcohol addiction, by one or both parents, was present in over half of the Significant Case Reviews (carried out when a child has died or been significantly harmed) between 2012 and 2015 and present in all cases where there was a death of an infant or pre-school child (4). Similar findings regarding risk and mortality have been reported in other countries (5–7). A follow up study in Glasgow, Scotland, of babies born to mothers with addiction issues found that 83% of children were discharged from the maternity unit to parental care, but 87% of these children were later taken into care at least once before the age of 10–12 years. Only 41% were in the care of their birth parent/s at 10–12 years of age (8).

Harm to children may be a direct result of exposure to substances prenatally, while other harms may be related to the multiple risk factors also associated with parental addiction including parental mental health issues (3, 9–11); domestic abuse (3, 9, 11, 12); poverty (3, 10, 13) and inadequate housing (11). These factors overlap in many situations to present a cumulative risk to parents' ability to adequately care for children (11, 14–16). Parents with addiction issues are therefore more likely to have their children removed from their care by social work services due to risk of harm or harm already caused (17, 18).

Not all child removals^2^ are to permanent placements. Almost a third of children taken into care in Scotland were returned to the care of their birth parents, with the average time to reunification being just over 9 months (19). Parental wellbeing is linked to child wellbeing (18), for example, parental stress and responsiveness have been associated with child cognitive development and prosocial behavior (20) and a recent systematic review found a preliminary link between parental mental health and wellbeing and intergenerational transmission of attachment but was unable to identify the mechanisms for this relationship (21).

However, removal of children also has the potential for harm, which may undermine the chances of reunification or increase the risk that children will be removed from their parents' care in the future. Parents and birth families report experiencing distress and a deterioration in their mental health following the removal of children. One study found roughly two thirds of birth parents and families reported symptoms or a diagnosis of depression which they felt was triggered or exacerbated by the removal, 26% experienced suicidal thoughts following the removal and roughly half of those reported an attempt to end their lives (22). In addition to reporting increased rates of suicide attempts and self-harm (23, 24), relapse or an increase in drug and alcohol use is common following removal (22, 23, 25, 26). Parents also reported experiencing strong negative emotions including anger, agitation, anxiety and sadness (23, 27–29).

In addition, a grief response is also experienced following the removal of children (26, 30–32). Disenfranchised grief is defined as “the experience of grief that is not openly acknowledged, socially validated or publicly observed” (33) and has been applied to mothers with children in the care system due to their grief response at the loss, the stigma of having a child removed and their own role in the removal (26). The lack of acknowledgment of this loss results in a lack of support or identifiable referral pathways for service input and can also lead to mothers developing beliefs about being undeserving of support (25). Birth mothers have reported feeling that their grief was not considered “legitimate” (23). While mothers who relinquished children experienced more grief symptoms than women whose child died and their grief reactions were more likely to become chronic and prolonged due to an inability to resolve their grief (34).

Low self-esteem is reported consistently following removal of children (22, 27). Mothers who have children removed have been described as “maternal outcasts”; mothers whose experiences fall outside of the normal expectations of motherhood (35). Mothers who have had children removed struggle with two main aspects of their identity—firstly, dealing with the stigma and shame attached to the removal of their child and their threatened identity as a “good parent” and secondly, difficulty maintaining an identity as a mother without a child in their care (26). Mothers with an addiction are also dealing with the additional stigma attached to having an addiction while being pregnant or as a mother (17, 36, 37).

Mothers who have had children removed describe the process and experience of removal as traumatic (23, 29). They describe the process as adversarial; with a focus on their weaknesses and little recognition of any strengths or positives in their parenting or relationship with their children (27). Parents reported feeling angry, humiliated and betrayed during the removal process (23, 26, 27).

Mothers with addiction issues are more likely than fathers to be primary carers (38) therefore they are more likely to experience removal of children and may be at greater risk of these subsequent issues following removal. In addition, service users in addiction and recovery services are predominately male (39) so services may not be focused on or aware of gender-specific issues that are more likely to have an impact on women, such as parenting issues or the impact of child removal into care (17, 39, 40). Exploring the impact of gender on child removal and associated factors could lead to increased understanding, improved mental health and reduced suicidality in women attending addiction services, new service developments and improvements in service delivery, especially for those women who are mothers.

We aimed to examine whether there were gender differences in the prevalence and patterns of child removal (i.e., individually or sibling groups) from parents, to examine the associations between child removal and parental factors (gender, age, substance use profile, mental health issues, and suicide attempts) and the relationship between removal and suicidality in parents attending an Alcohol and Drug Recovery Service in Scotland.

## Materials and Methods

### Procedure

This study was conducted within one sector of an Alcohol and Drug Recovery Service in Glasgow, Scotland with roughly 3,000 active service users. To access the service individuals need to have moderate to severe addiction issues and complexity or risk (such as physical or mental health issues, childcare, criminal justice involvement).

Data were gathered on ~25% of randomly selected service users as detailed in Supplementary Table 1. Due to the high levels of disengagement from the service, staff were randomized rather than service users and 100% of staff provided a copy of their caseload. The Research and Innovation Department advised that this study did not need to go to ethics committee due to the use of routinely collected patient data. Therefore, the study was registered with and approved by the Alcohol and Drug Recovery Service Clinical Effectiveness Group. Service users consent at assessment that their routinely collected data can be used anonymously for research and audit purposes.

Staff were randomly selected in June 2015 and data were collected from electronic records from June 2015 to June 2017. Electronic records included the Scottish Morbidity Record 25 (SMR25), which are compulsory data returns completed at assessment (Version A) and annually (Version B) in Scottish Alcohol and Drug Recovery Services, and clinical case notes. Staff interviews were conducted between September 2015 and June 2017. A proforma was created for each format (SMR25, case notes and interviews) for data collection and categorization. Initially the SMR25 forms were reviewed, then the clinical case notes. Once these were completed for the full caseload, interviews were arranged with staff members. Case notes and staff interviews allowed for the cross-checking of the SMR25 data and collecting any missing data.

### Data Collection

Data were collected under the following headings: (1) Service user characteristics; (2) Child characteristics; (3) Mental health; and (4) Suicide.

(1) Service user characteristics
  SMR25—Gender, age, ethnicity, substance use profile (treatment provided for drugs only; alcohol only; alcohol and drugs).
  Case notes—Used for missing data.
(2) Child characteristics
  SMR25—Number of children, number of children removed by Local Authority.
  Case notes—Missing data and pattern of removal (one child or all children at one time; two groups or a group and a single child removed at different times; series of individual removals).
  Staff interviews—Used for missing data.
(3) Mental health
  SMR25—Reviewed questions on current or history of mental health issues and prescribed medication for mental health issues.
  Case notes—Reviewed for any mention of mental health diagnosis, contact with mental health services, requests for mental health assessment or a referral to mental health services, reported use of psychotropic medication, inpatient admissions to mental health units/wards.
  Staff interviews—Asked if service user had current or history of mental health issues.
(4) Suicide
  SMR25—Reviewed question on ever attempted suicide.
  Case notes—Reviewed for any mention of suicide attempts.
  Staff interviews—Asked if service user had ever attempted to take their own life.

### Statistical Analysis

Data analysis using SPSS (version 28.0.0.0) was conducted to explore any differences between genders in demographic factors and in prevalence and patterns of child removal. Binary logistical regression was conducted to examine risk factors associated with child removal. Of the 736 service users selected for the study, parents who had no children removed (*n* = 287) were compared with parents who had experienced removal of children (*n* = 158). Factors examined were age, gender, substance use profile, mental health issues and suicide attempts. Ethnicity was excluded due to the lack of variability in this sample. The analysis was then repeated for each gender. Chi-squared analysis was used to further explore the relationship between suicidality and child removal.

## Results

### Descriptive

The interviews and caseload reviews of the 8 nurses and 12 social care workers produced data on 736 (~24.5%) of service users. Table 1 illustrates the demographic information for the total sample plus each gender. The sample was 66% male and 97% White Scottish. Substance use profiles were similar across genders, but women were significantly younger and more likely to have a current or history of mental health issues and suicide attempts.

**Table 1:** Demographic information.

**Demographic factors**	**Females** **(*N* = 250)** ***N* (%)**	**Males** **(*N* = 486)** ***N* (%)**	**Total Sample** **(*N* = 736)** ***N* (%)**
Age^*^			
Mean (years)	40.3	44.1	42.8
Range (years)	15–78	21–78	15–78
**Substance use profile**			
Drugs only	144 (57.6)	274 (56.4)	418 (56.8)
Drugs and alcohol	66 (26.4)	105 (21.6)	171 (23.2)
Alcohol only	40 (16)	107 (22)	147 (20)
Current or history of mental health issues^*^			
Yes	176 (70.4)	236 (48.6)	412 (56)
History of suicide attempts^*^			
Yes	116 (46.4)	100 (20.6)	216 (29.3)

* *Indicates significant difference between genders (age p < 0.001; mental health p < 0.001; suicide p < 0.001)*.

### Prevalence and Patterns of Child Removal

Data were analyzed to investigate the prevalence of removal of children. Patterns of removal (one episode of a single child or a sibling group; two removals of sibling groups or a sibling group and an individual child at a separate time; or repeated individual removals) were also analyzed and are reported in Table 2.

**Table 2:** Prevalence and patterns of child removal.

**Removal**	**Mothers** **(*N* = 196)** ***N* (%)**	**Fathers** **(*N* = 266)** ***N* (%)**	**All parents** **(*N* = 462)** ***N* (%)**
Children removed^*^			
Median	2	1	1
Range	0–6	1–4	1–6
One episode of removal (child or sibling group)	73 (65.8)	35 (74.5)	108 (68.4)
Two episodes of removals involving groups	4 (3.6)	4 (8.5)	8 (5.1)
Series of removals of individual children^*^	25 (22.5)	2 (4.3)	27 (17.1)
Prevalence^*^	111 (56.6)	47 (17.7)	158 (34.2)

* *Indicates significant difference between genders (children removed p < 0.001; pattern p =‘0.014; prevalence p < 0.001)*.

There was a significant difference in prevalence between genders with removal being more likely from mothers than fathers. Mothers had greater number of children removed than fathers. There was also a significant difference in removal patterns with mothers being more likely to experience repeated individual removals.

### Child Removal and Relationships With Age, Gender, Substance Use, Mental Health, and Suicide Attempts

Table 3 illustrates the odds ratios for the associations between each factor and child removal.

**Table 3:** Factors associated with the removal of children^*^.

**Factors**	***B***	**S.E**.	**Wald**	**df**	***p***	**OR**	**95% C.I**.
Age	−0.06	0.01	20.76	1	<0.001	0.95	0.92–0.97
Gender	1.78	0.22	66.58	1	<0.001	5.91	3.85–9.05
Substance use	0.78	0.28	7.68	1	0.006	2.19	1.26–3.8
Mental health issues	0.51	0.21	6.11	1	0.013	1.66	1.11–2.49
Suicide attempts	1.06	0.21	25.13	1	<0.001	2.89	1.91–4.38

* *Each association takes the other factors into account*.

Parental age was significantly negatively associated with removal and with each increasing year parents were less likely to have their child or children removed. Mothers were nearly six times more likely than fathers to experience removal. Parents with drug or drug and alcohol addictions were more than twice as likely to experience removal than those with only alcohol addictions. Parents with mental health issues were nearly 70% more likely to have children removed and parents who had attempted suicide were nearly three times more likely to have children removed.

To examine the impact of gender on removal, the analysis was repeated separately for each gender. For women, younger age, drug/drug and alcohol use, mental health issues and suicide attempts continued to be significantly associated with child removal. No factors were significantly associated with child removal in fathers.

**Figure 1:**
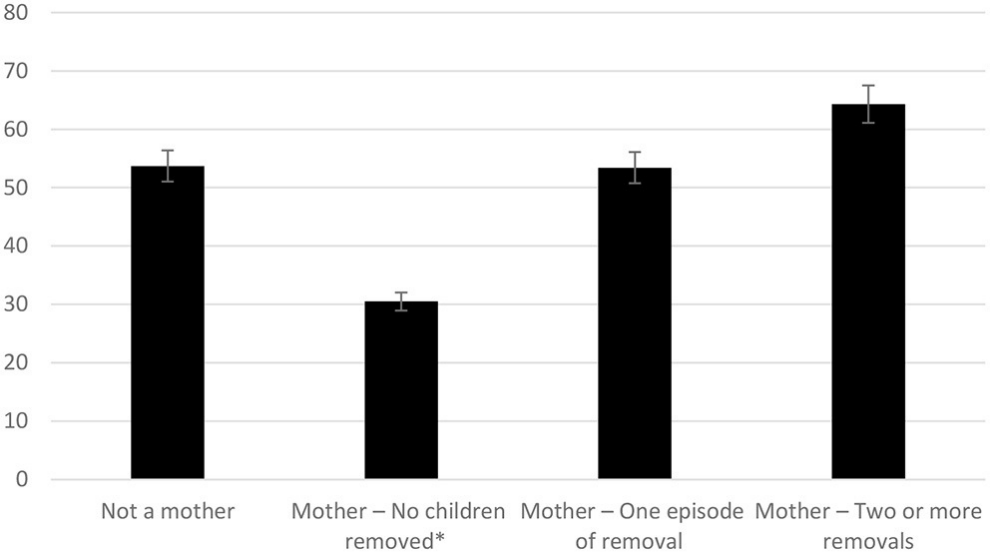
Prevalence of suicide attempts (%) across different removal groups in mothers. *Indicates significant difference between groups (*p* = 0.003).

To further explore the relationship between suicidality and child removal, chi-squared analysis compared rates of suicidality across removal groups (not a mother, mother no removals, mother one episode of removal, mother more than one episode of removal). Due to the small number of group removals; data were recategorised to one episode of removal or more than one episode of removal. Figure 1 highlights the increase in prevalence of suicide attempts as the number of child removals increases. Mothers who had not experienced removal were significantly less likely to have attempted suicide than women who were not mothers, and mothers who had experienced removal.

## Discussion

There were stark gender differences found in our study when it came to prevalence and patterns of child removal. Women make up 34% of the service but 78% of these women are mothers while 55% of men were fathers. Women were more likely to be parents than men and more than half of female service users who were mothers had one of more of their children removed compared with less than a fifth of fathers. Mothers were almost six times more likely to experience removal than fathers. Some of which may be explained by the high rates (~92%) of female-headed single parent families in Scotland (41). It is also important to note that while the majority of removals across both genders involved a single episode of removal, some of these parents are still of reproductive age with the potential to have further children and experience further removals.

Previous research has shown that women are more likely to have their children removed than fathers, even when fathers are perpetrators of similar levels of abuse or neglect (42). Women using addiction and recovery services report experiencing barriers accessing services and having additional needs related to their family and carer responsibilities, relationships, and mental health issues (39, 43). When caring for children, women are more likely to experience isolation due to higher rates of domestic and interpersonal abuse which results in less support with parenting (44, 45). There are recommendations that gender specific issues should be acknowledged in addiction and recovery services including the need for single gender support groups, interventions related to trauma, relationships and parenting and the provision of childcare (17, 43, 46). Our findings add further support to the recommendations for the provision of childcare and parenting interventions with the high rates of women in this service having children and concerns about parenting and risk due to the prevalence of child removal. Foster care provided by Glasgow City Council Social Work costs roughly £500 per child/week and is more expensive when provided by external providers (8). Therefore, providing parenting interventions has the potential not only to reduce costs but also to reduce risk and save lives of women and their children.

The lack of awareness and acknowledgment of gender specific issues on the part of staff may result in mothers receiving treatment for their addiction without consideration of how the experience of being a mother, their feelings about the impact of their addiction on their children and the impact of removal of children may be linked to their recovery, or lack of. Indeed, we found a significant relationship between removal of children and suicidality. If services fail to acknowledge or ask about child removal, then they are constantly failing women with addiction issues by using an individualized rather than a family focused approach which risks excluding the most vulnerable women and their families and perpetuates further harm. Therefore, we recommend that services ask all female service users about children and child removal and do not just focus on current children in their care. While current child information is essential for child protection and welfare, the links found between child removal and suicide mean any information related to child removal needs to be included as part of the mother's risk assessment and treatment plan. This may also highlight if additional support is needed during and after removal or at significant dates such as date/s of removal and children's birthdays. Support may involve attendance at meetings with the Local Authority, referrals for mental health treatment, supporting women to make and accompanying them to appointments and encouragement to engage with peer recovery support groups. In addition, staff should also monitor for change in frequency or pattern of drug/alcohol use, mood, increase in suicidality or self-harm and withdrawal from usual routines or support systems as this might indicate increased risk.

As this is a cross-sectional study, we cannot infer the direction of causality: women with more severe mental health issues and greater suicidality might be more likely to have their children removed, but it is also possible that suicide attempts followed removal of children. Future longitudinal studies will be required to evidence this, but the link underscores the vital need to understand the relationship between parent factors and child factors if we are to better support recovery from addiction and the wellbeing of children.

This study identified a group of parents who had multiple children individually removed from their care; who were more likely to be mothers than fathers. Previous research has also indicated that mothers are more likely to experience repeated individual removals (35, 47). Our study identified the group at the highest risk of having their children removed as younger women who had drug and mental health issues and who had attempted to take their own lives: this supports previous findings linking younger maternal age to risk of repeated removals (47) and younger age, mental health issues and substance use with involvement in care proceedings (14).

Stigma may have a role in explaining why drug use, as opposed to alcohol use, was a risk factor for removal. Alcohol use is more socially acceptable (48) and risk to children from alcohol might therefore attract less stigma than drug use despite the fact that prenatal alcohol use is associated with more harm than prenatal drug use (49). Women report experiencing, or perceiving they experience, greater stigma than men due to their addiction issues especially when mothers or pregnant (17, 39, 50, 51). The fear of increased stigma and concerns about the removal of children can act as a barrier to pregnant women or mothers accessing addiction and recovery services (51) which delays treatment, placing these women and their children at increased risk of harm.

These findings on removal risk factors support previous research indicating that parental addiction commonly occurs within a constellation of other risk factors (14, 16) that are cumulative (15, 16). This complexity suggests that interventions aimed at reducing harm to children by focusing solely on parental addiction may not improve outcomes and may actually worsen outcomes. Instead, we suggest a public health approach is needed focusing on early intervention with high-risk families, taking a holistic view to target the multiple areas of support needed by these families and the cyclical effects that may occur when addiction affects child outcomes, which further affects parental mental health and the success of addiction and recovery services in improving adult outcomes. The divide between social work, adult mental health and children's health services makes implementation science challenging and we need to bridge the gap between these services through partnership working. We suspect this would be viewed as challenging by services but there are examples of good partnership working which acknowledge the complexity, challenges and benefits that this style of working brings (52).

Due to engagement issues the sample was obtained by randomly sampling staff rather than service users. Hundred percent of staff provided a copy of their caseload. A strength of this study is the sample size and its representativeness of the wider service. It also includes service users at all stages of treatment from assessment onwards rather than just those who completed treatment. These findings are likely to be generalizable to other addiction and recovery services but may not be fully generalizable to other geographical areas, especially those with greater ethnic diversity. In addition, this sample may not be representative of parents with addiction issues who are not engaged with services; such as parents who do not meet the criteria for the service due to milder levels of addiction issues, including those who are engaging with community organizations such as 12 step groups or third sector organizations, and parents who are actively trying to avoid engaging with services. Another limitation is the use of self-report information and routinely collected data about child removal and mental health issues as this may be underreported or minimized; although some of this data was corroborated by health and social work records.

Because only one researcher was given permission to access the data, no reliability checking by a second rater was possible. Additionally, data was only accessible from parent's records and not their children's. As a result, it was not possible to assess if parents were primary carers before removal occurred. Therefore, our data on parents who have experienced removal may include a subgroup of parents who were not primary carers prior to removal. Also, it was not always possible to access information on when children were removed. While we were able to collect data on quantity and frequency of alcohol and/or drug use at the time of data collection, this may not be an accurate reflection of their addiction at the time of removal. Therefore, we categorized service users depending on whether they were receiving treatment for drug use only, alcohol use only or drug and alcohol use. We collected data on suicide attempts and suicidal behavior may be underrepresented if service users did not disclose attempts to end their life to their care manager. Similarly, the data does not capture other risk markers such as self-harm and recurrent suicidal ideation.

## Conclusion

We have shown that mothers with addiction issues are six times more likely to have their children removed than fathers and these mothers are significantly more likely to have made attempts to end their lives. We have evidenced the complexity of the relationship between parental factors and the removal of children from parental care, implicating the mental health and suicidality of parents in addiction and recovery services. This makes it clear that these findings have implications for both health and social care services and highlight the importance and value of partnership working. This is an urgent issue with has an impact on mortality, wider society, and children's life chances.

While it is clear that addiction of parents can have a serious effect on children and result in the removal of children, the removal of children is having a serious effect on parents, which may in turn further exacerbate their addiction and further affect children who may return to their care and/or any future children they might have. This cyclical process is in dire need of further investigation, particularly qualitative work with parents in addiction and recovery services to better understand how unmet needs and child removal are affecting both parents and children.

## Data Availability Statement

The raw data supporting the conclusions of this article will be made available by the authors, without undue reservation.

## Ethics Statement

Ethical approval was not provided for this study on human participants because data was collected from routinely collected data. NHS GG&C R&I department confirmed that NHS Ethics was not needed and recommended review by NHS Clinical Effectiveness Committee who provided approval. The participants provided their written informed consent to participate in this study.

## Author Contributions

LR conducted the acquisition, analysis, and interpretation of data for the work. LR, RG, FT, and HM made substantial contributions to the conception or design of the work, drafting the work or revising it critically for important intellectual content, provided approval for publication of the content, and agree to be accountable for all aspects of the work in ensuring that questions related to the accuracy or integrity of any part of the work are appropriately investigated and resolved. All authors contributed to the article and approved the submitted version.

## Funding

This work was supported by University of Glasgow.

## Conflict of Interest

The authors declare that the research was conducted in the absence of any commercial or financial relationships that could be construed as a potential conflict of interest.

## Publisher's Note

All claims expressed in this article are solely those of the authors and do not necessarily represent those of their affiliated organizations, or those of the publisher, the editors and the reviewers. Any product that may be evaluated in this article, or claim that may be made by its manufacturer, is not guaranteed or endorsed by the publisher.

## Footnotes

^1^Parental addiction includes the terms parental substance abuse and misuse and covers both alcohol and drugs.

^2^Child removal refers to children removed from their parents and placed in alternative care by the Local Authority and does not include informal agreements.
