# Supplementary material for: Gender, Addiction, and Removal of Children Into Care
Source: Front Psychiatry. 2022 Jun 2;13:887660. doi: 10.3389/fpsyt.2022.887660 (PMC9201045; doi:10.3389/fpsyt.2022.887660)
Supplement: Supplementary file 1 [file Table_1.DOCX]

Supplementary Material

Supplementary Table 1: Sampling and data collection process

| **Stage** | Procedure |
| --- | --- |
| 1 | Staff members were excluded if they were currently off work for an extended period of time (such as maternity or long term sick leave), did not have a caseload (such as team leads or students) or were co-workers (such as health care workers, medics and the blood borne virus nurse). |
| 2 | Remaining staff were split into two groups of 25 nurses and 40 social care workers. |
| 3 | Surnames were entered into two SPSS worksheets. |
| 4 | SPSS generated a random sample of 6 nurses and 10 social care workers (~25%). |
| 5 | Each member of staff was approached by the researcher and provided with information about the study. |
| 6 | All (100%) members of staff provided the researcher with a copy of their full caseloads. |
| 7 | Each service user’s unique ID number was recorded in an Excel database to prevent duplication. |
| 8 | Service users were only included if the member of staff was their care manager rather than a co-worker for a brief piece of work (such as a physical or mental health assessment). |
| 9 | Electronic records data were collected before speaking to the worker in all cases. |
| 10 | Data from each member of staff’s caseload was fully gathered before starting with the next member of staff. |
| 11 | Due to varying caseloads (due to role or part time working) and exclusions of service users (as a result of duplication or co-working) the original sampling process failed to generate a sample of ~25% of service users. Staff previously included in the study were removed from the original list of surnames in SPSS and stages 1 to 11 were repeated to generate an additional 2 nurses and 2 social care workers and a sample of ~24.5%. |
